# Adjuvant Probiotics of *Lactobacillus salivarius* subsp. *salicinius* AP-32, *L. johnsonii* MH-68, and *Bifidobacterium animalis* subsp. *lactis* CP-9 Attenuate Glycemic Levels and Inflammatory Cytokines in Patients With Type 1 Diabetes Mellitus

**DOI:** 10.3389/fendo.2022.754401

**Published:** 2022-03-01

**Authors:** Chung-Hsing Wang, Hung-Rong Yen, Wen-Li Lu, Hsieh-Hsun Ho, Wen-Yang Lin, Yi-Wei Kuo, Yen-Yu Huang, Shin-Yu Tsai, Hung-Chih Lin

**Affiliations:** ^1^ Division of Medical Genetics, Pediatric Endocrinology & Metabolism, China Medical University Children’s Hospital, China Medical University, Taichung, Taiwan; ^2^ School of Medicine, College of Medicine, China Medical University, Taichung, Taiwan; ^3^ Department of Chinese Medicine, China Medical University Hospital, Taichung, Taiwan; ^4^ Research Center for Traditional Chinese Medicine, China Medical University Hospital, Taichung, Taiwan; ^5^ Chinese Medicine Research Center, China Medical University, Taichung, Taiwan; ^6^ Department of Biotechnology, Asia University, Taichung, Taiwan; ^7^ Research and Development Department, Bioflag Biotech Co., Ltd., Tainan, Taiwan; ^8^ Division of Neonatology, China Medical University Children’s Hospital, Taichung, Taiwan; ^9^ School of Chinese Medicine, China Medical University, Taichung, Taiwan; ^10^ Asia University Hospital, Asia University, Taichung, Taiwan

**Keywords:** T1DM, probiotic, gut microbiota, glycemic levels, immune cytokines

## Abstract

**Introduction:**

Type 1 diabetes mellitus (T1DM) is characterized by autoimmune destruction of pancreatic β cells. Previous study has discovered that probiotic strains residing in the gut play essential roles in host immune regulation. However, few clinical results demonstrated probiotic would actually benefit in attenuating glycated hemoglobin (HbA1c) along with inflammatory cytokine levels of the T1DM patients and analyzed their gut microbiota profile at the same time. In this clinical trial, we evaluated the therapeutic efficacy of probiotics on HbA1c along with inflammatory cytokine levels of T1DM patients to determine an alternative administration mode for T1DM medication. The probiotics changed T1DM gut microbiota profile will be measured by next-generation sequencing (NGS).

**Research Design and Methods:**

A randomized, double-blind, placebo-controlled trial was performed at China Medical University Hospital. T1DM patients between 6 and 18 years of age were enrolled. 27 patients were administered regular insulin therapy plus capsules containing probiotic strains *Lactobacillus salivarius* subsp. *salicinius* AP-32, *L. johnsonii* MH-68, and *Bifidobacterium animalis* subsp. *lactis* CP-9 daily for 6 months, and 29 patients were administered insulin therapy without extra probiotic supplement as placebo group. The variations of fasting blood glucose and HbA1c in these patients were analyzed. In addition, serum levels of inflammatory cytokines and anti-inflammatory cytokine were assessed using enzyme-linked immunosorbent assay. Patients’ stool microbiota were all subjects to NGS analysis.

**Results:**

NGS data showed elevated populations of *Bifidobacterium animalis, Akkermansia muciniphila* and *Lactobacillus salivarius* in the gut of patients with T1DM who were taking probiotics. Patients with T1DM who were administered probiotics showed significantly reduced fasting blood glucose levels compared with the before-intervention levels. The HbA1c levels of the patients also improved after administration of probiotics. The concentrations of IL-8, IL-17, MIP-1β, RANTES, and TNF-α were significantly reduced and were associated with an increased TGF-β1 expression after probiotic intervention. The persistence effect of glycemic control and immunomodulation were observed even 3 months after discontinuation of the probiotics.

**Conclusions:**

Here, we found that conventional insulin therapy plus probiotics supplementation attenuated T1DM symptoms than receiving insulin treatment only. Probiotics supplementation with insulin treatment changed gut microbiota and revealed better outcome in stabilizing glycemic levels and reducing HbA1c levels in patients with T1DM through beneficial regulation of immune cytokines.

**Clinical Trial Registration:**

ClinicalTrials.gov, identifier NCT03880760.

## Introduction

Type 1 diabetes mellitus (T1DM) is a chronic autoimmune disease, wherein pancreatic beta cells are attacked and disrupted by abnormal immune response (1). The pancreatic beta cells produce insulin and regulate glycemic homeostasis in the human body (2). Damage to the beta cells causes insulin insufficiency, and the elevation and accumulation of blood glucose leads to stroke, heart diseases, damage to the nervous system, impaired cognition, kidney failure, poor vision, and numbness in limbs (3, 4). An estimated 1,106,500 individuals aged 0–19 years worldwide are diagnosed as having T1DM, which accounts for 5%–10% of all patients with diabetes with >85% of them being young patients (5, 6). Insulin injection is the major clinical treatment for T1DM, but a cure for T1DM has not yet been reported (7). Therefore, understanding the pathogenesis of T1DM and prevention of its onset are the main strategies in the investigation of this disease (8).

Some researchers have reported that autoimmune reactions against beta cells may come from the activation of the immune system in genetically susceptible individuals, which is triggered by environmental factors that bear epitopes similar to those expressed by the beta cells. Several mechanisms, such as viral infections or other triggers (antigens from diet) were considered to initiate a hyperactive response toward β-cells (5, 9, 10). Other studies have publicized that 10-30% of patients with T1DM develop other autoimmune diseases (11). The clinicopathological mechanism of celiac disease is the one similar to T1DM, there is a high probability that both diseases have comorbidities. Interestingly, while celiac disease composes a proportion of the T1DM population in the Western countries (11), which is less common or nonexistent in the Asian population (12).

Studies have reported a link between gut microbiota and T1DM. Decreased population of Firmicutes and increased population of Bacteroidetes have been observed in most patients with T1DM. At the genus level, T1DM children contained higher population of *Veillonella*, *Clostridium*, and *Bacteroides* in intestine, whereas the healthy children had higher populations of *Prevotella, Blautia coccoides/Eubacterium* rectale group, *Lactobacillus*, and *Bifidobacterium* (13, 14). The connection between host immune cells and gut microbiota may also influence the pathogenesis of T1DM. Metabolites secreted by the gut microbes can regulate the immune response and slow down the development of process of T1DM (15, 16). In other words, dysbiosis of the gut microbiota may incite and accelerate the pathogenesis of T1DM (17).

The microbiota modulators and probiotics help to maintain a healthy homeostasis of gut microbiota, gut membrane integrity, and permeability and also upregulate anti-inflammatory cytokines such as transforming growth factor-β (TGF-β) while downregulating proinflammatory cytokines such as TNF-α (18). The functions of some microbial metabolites, including short-chain fatty acids and lactic acids, on modulation of innate immune response have also been reported (19). Thus, probiotics could be a potential regulator of T1DM and could help slow disease progression *via* the gut microbiota-immune axis.

Probiotic, which was defined as: “live microorganisms which when administered in adequate amounts confer a health benefit on the host” (20). However, few studies have demonstrated the benefits of probiotics to patients with T1DM. Probiotic strains *L. salivarius* subsp. *salicinius* AP-32, *L. johnsonii* MH-68, and *B. animalis *subsp. *lactis* CP-9 were screened by their ability of consuming glucose *in vitro (* 21 *).* In addition, *L. salivarius* subsp. *salicinius* AP-32 could enhance the expression of glucose transporter 2 in human intestinal epithelial cells and played a major role in regulating glucose metabolism and blood lipid levels in mice models of T2DM (21). Moreover, an *in vivo* study also indicated that *L. johnsonii* MH-68 and *L. salivarius* subsp. *salicinius* AP-32 reduced the levels of inflammatory chemokines (22).

The aim of this study is to improve glycemic control through reduction of inflammatory cytokines by means of daily use of probiotic strains. The strains used were *L. salivarius* subsp. *salicinius* AP-32, *L. johnsonii* MH-68, and *B. animalis* subsp. *lactis* CP-9. Furthermore, the feces were tested for microbiota load to ensure that the consumption of the aforementioned probiotics was effectively colonized in the recipients’ gut systems. The results of this study might provide a novel probiotic combination to ameliorate or even prevent the clinical symptoms of the chronic autoimmune disease, T1DM.

## Research Design and Methods

Patients with age of onset between 6 and 18 years and were diagnosed with T1DM (i.e., fulfilling 2 of the 3 characteristics of the following: 1) ketoacidemia, 2) insulin-related autoantibodies, and 3) insufficient insulin secreting capacity) were included in the study (23, 24). Given that these patients are longstanding diabetic patients who have already experienced the honeymoon periods, they have gone through thorough education regarding diabetes and diet. Patients with significant heart, kidney, and liver diseases; those diagnosed as having immunodeficiency and low immune function; those using probiotic-related products or those who had taken probiotics for more than 1 month; those who were taking antibiotics or stomach and intestinal drugs at the start of the trial; and those diagnosed as having allergies to probiotics were excluded. A total of 64 patients were initially recruited, 5 of whom withdrew from the study.

Thus, 59 patients were enrolled. *Via* computer-generated random numbering with double blinding, 27 were assigned to the probiotic group (female/male = 14/13) and 32 were assigned to the placebo group, out of which 3 withdrew due to loss to follow-up and extreme fluctuations in blood sugar (female/male = 10/19). Finally, a total of 56 patients were included for analysis (**Supplemental Figure S1**). Patients in the probiotic group were instructed to consume 1 × 10^10^ colony-forming units (CFU)/day of mixed probiotics *(L. salivarius* subsp. *salicinius* AP-32, *L. johnsonii* MH-68, and *B. animalis* subsp. *lactis* CP-9) for 6 months, during which their insulin regimens were given as per existing protocol (**Table 1**) (25), and 3-month follow-up (26). The fasting blood glucose (Glucose AC), glycated hemoglobin (HbA1c), immune cytokine concentration, and fecal bacterial phase changes before and after intervention were compared between the groups.

**Table 1:** Baseline demographic and laboratory characteristics of patients with T1DM.

Parameter	Probiotics (n = 27)	Placebo (n = 29)	P-value
Age (years)	14.1 ± 5.1	14.3 ± 4.6	0.824
Sex (F/M)	14/13	10/19	0.280
T1DM duration (months)	74.4 ± 53.6	77.9 ± 46.9	0.640
Insulin regimen (N)			
BID AC	9	6	0.370
TID AC	2	1	0.605
QID AC	16	22	0.254
Insulin dosage (U/kg/day)	0.8 ± 0.3	0.8 ± 0.3	0.889
Glucose AC (mg/dL)	185.4 ± 41.5	172.2 ± 62.6	0.131
HbA1c (%) (mmol/mol)	9.3 ± 0.8 78.0 ± 8.9	9.5 ± 1.9 79.9 ± 21.2	0.883 0.928
TNF-α (pg/mL)	52.5 ± 59.7	54.0 ± 56.8	0.812
IL-8 (pg/mL)	398.2 ± 233.4	477.7 ± 222.9	0.093
IL-17 (pg/mL)	26.8 ± 34.1	19.5 ± 24.5	0.441
MIP-1β (pg/mL)	113.9 ± 75.5	130.5 ± 86.9	0.549
RANTES (pg/mL)	447.9 ± 71.4	411.0 ± 69.0	0.068
TGF-β1 (pg/mL)	5624.2 ± 1984.6	6554.3 ± 1759.3	0.068

BID AC, Twice a day before meals; TID AC, Three times a day before meals; QID AC, Four times a day before meals and bedtime.

Placebo group: T1DM patients receive regular insulin treatment but no additional probiotic supplementation.

Probiotic group: T1DM patients receive regular insulin treatment with additional probiotic supplementation.

### Probiotic Strains and Cultivation

Active probiotic strains *L. salivarius* subsp. *salicinius* AP-32, *L. johnsonii* MH-68, and *B. animalis* subsp. *lactis* CP-9 were obtained from Bioflag Biotech Co., Ltd. (Tainan, Taiwan). AP-32 and MH-68 strains were isolated from human gut and CP-9 strain was isolated from breast milk. The deposition numbers for *L. salivarius* subsp. *salicinius* AP-32, *L. johnsonii* MH-68 and *B. animalis* subsp. *lactis* CP-9 were CCTCC-M2011127, CCTCC-M2011128, and CCTCC-M2014588, respectively. The above-mentioned strains are preserved in China Center for Type Culture Collection (abbreviated as CCTCC) and China General Microbiological Culture Collection Center (abbreviated as CGMCC).

De Man, Rogosa, and Sharpe (MRS) broth was used to culture *Lactobacillus* spp., and MRS broth supplemented with 0.05% cysteine was used to culture *Bifidobacterium* spp. The selected probiotics were incubated at 37°C under anaerobic conditions for 20 hours and subjected to lyophilization. The viability of dry bacteria was determined by analyzing the CFU. The dosage of the mixture of AP-32, MH-68, and CP-9 strains was 5 × 10^9^ CFU/capsule. The mixing ratio of the three probiotics was 1:1:1.

### Measurement of Serum Glucose (AC)

The patients underwent blood tests in the department of laboratory medicine in China Medical University Hospital, Taichung, Taiwan. The fasting blood glucose values were detected by Optium Xceed Glucometer (Abbott Diabetes Care Inc., Alameda, CA, USA) by endpoint detection colorimetry. Glucose AC was measured and analyzed before intervention, 6 months after intervention initiation, and 3 months after stopping the intervention in both the groups.

### Measurement of Serum HbA1c Level

Glycated hemoglobin (HbA1c) value was measured using the HbA1c HPLC Assay Kit (Eagle Biosciences, Nashua, NH, USA, catalog number: A1C31-H100) with high-performance liquid chromatography (HPLC, PerkinElmer Series 200). The HbA1c was measured and analyzed before intervention, 6 months after intervention initiation, and 3 months after stopping the intervention in both the groups.

### Measurement of Cytokine Levels by Enzyme-Linked Immunosorbent Assay (ELISA)

Extra blood left after the above tests was used to detect several inflammatory or anti-inflammatory cytokines before intervention, 6 months after intervention initiation, and 3 months after stopping the intervention. After centrifugation, the serum was refrigerated at −80°C until use for ELISA. The inflammatory cytokines used for ELISA test were as follows: TNF-α (Thermo Scientific, Carlsbad, CA, USA), IL-8 (Thermo Scientific, Carlsbad, CA, USA), IL-17 (R&D Systems, Minneapolis, Minnesota, USA), MIP-1β (PeproTech, Cranbury, NJ, USA), and RANTES (R&D Systems, Minneapolis, Minnesota, USA). The anti-inflammatory cytokine used was TGF-β1 (Thermo Scientific, Carlsbad, CA, USA). All samples were measured by the commercial protocol at least in triplicate.

### Detection of Gut Microbiota Using NGS

The fecal samples of the patients were collected before and after taking probiotics, and immediately refrigerated at −80°C. Once the stool samples had thawed, the DNA (approximately 50–100 mg) was extracted by Quick-DNA™ fungal/bacterial micro-preparation reagent (ZYMO Research, Irvine, CA, USA). The DNA sample was then diluted to 5 ng/μL in sterile water and provided to Phalanx Biotech Co., Ltd., Taiwan for quality inspection on 1% agar gel electrophoresis. The DNA fragments (16S rRNA, 16S V3-V4) were amplified by PCR through special primers using the 2× KAPA HiFi HotStart ReadyMix Kit (KAPA Biosystems, USA). The DNA product of 460 base pair was selected for the next experiment. All amplified DNA samples were purified using AMPure XP beads (Beckman Coulter Genomics, USA). The purified DNA was used to generate a sequenced sample library using Illumina Nextera XT Index kit (Illumina, USA). The sequenced sample library was evaluated using Qubit 2.0 Fluorometer (Thermo Fisher Scientific, USA) and Agilent Bioanalyzer 2100 system (Agilent Technologies, Inc., USA). Finally, this sequencing library was sequenced on the Illumina MiSeq platform.

The adaptors were first trimmed from the sequencing reads. All paired-end reads were merged by PEAR v0.9.10. QIIME v.2 as the quality parameter was used to demultiplex and filter at Q20. PCR generated amplicons with chimeric sequences were detected by USEARCH. 99% similarity sequence of clustering operational taxonomic units (OTU) were analyzed by QIIME v.2 with SILVA v.132 database. Sequences that appeared once (singletons) or failed mapped to database were discarded. The taxonomy was classified after annotating OTU.

### Statistical Analysis

Based on sample size calculation of previous study, the mean value of HbA1c at before-intervention was evaluated at 8.4% with a standard deviation of 1.3% for both groups (27). The sample size was calculated for two-tailed *t* test comparing two groups. In this per-protocol analysis, a clinically significant reduction of 1% in HbA1c was employed given that previous studies reported that every 1% reduction in HbA1c reduces the relative risk by 37% for microvascular complications, by 21% for diabetes-related deaths, and 14% for myocardial infarction (28, 29). For a power of 80% and a type 1 error of 0.05, the number of subjects needed for each arm of the study is 27. Assuming a drop-out rate of 10%, approximately 30 subjects was required for each arm in the study. The values of continuous variables are expressed as mean ± standard deviation. Fisher’s exact test was used to compare categorical variables. Wilcoxon signed-rank test and Mann–Whitney *U* test were used to compare the differences between variables. For NGS analysis, the permutation method was used to test the taxonomic groups (phyla, phylum, order, family, genus, and species). Statistical analysis was performed using SPSS 12 (IBM, USA). *P* < 0.05 was considered statistically significant. Changes in the microbiota were generated by GraphPad Prism 8 (GraphPad Software, San Diego, CA, USA).

## Results

### Patient Clinical Characteristics Before Probiotic Intervention

The clinical characteristics of all patients before intervention are shown in **Table 1**. A total of 27 patients with T1DM were assigned to the probiotic group and 29 were assigned to the placebo group (**Supplemental Figure S1**). The average age of the patients in the probiotic and placebo groups was 14.1 and 14.3 years, and T1DM duration were 74.4 and 77.9 months (**Table 1**). Among 27 patients under probiotic treatment, 9 patients were injected insulin twice a day before meals (BID AC), 2 patients were injected insulin three times a day before meals (TID AC) and 16 patients were injected insulin four times a day before meals (QID AC). Among 29 patients in the placebo group, 6 patients were injected insulin before meals BID AC, 1 patient was TID AC, and 22 patients were QID AC. The dosage of injected insulin was analogous in two groups (0.8 U/kg/day). The average Glucose AC was 185.4 (mg/dL) in probiotic group and 172.2 (mg/dL) in placebo group, and the average HbA1c of the two groups was 9.3% (78.0 mmol/mol) and 9.5% (79.9 mmol/mol), respectively. The levels of six cytokines were measured for patients in the probiotic and placebo groups. The initial average values were as follows: the average TNF-α level was 52.5 and 54.0 pg/mL, IL-8 was 398.2 and 477.7 pg/mL, IL-17 was 26.8 and 19.5 pg/mL, MIP-1β was 113.9 and 130.5 pg/mL, RANTES was 447.9 and 411.0 pg/mL, and TGF-β1 was 5624.2 and 6554.3 pg/mL in the probiotic and placebo group, respectively. There was no statistically significant difference in any of the diagnostic items between the two groups before intervention (**Table 1**).

### Elevated Population of *B. animalis* and *L. salivarius* in the Gut of Patients With T1DM After Taking Probiotics

**Figure 1:**
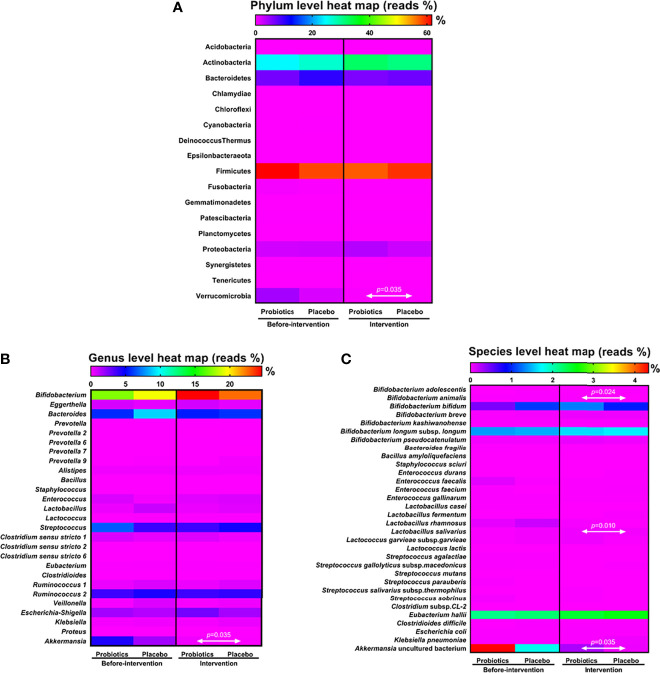
Analysis of microbiota dispersion (%) among patients with T1DM. The data present microbiota changes at the phylum **(A)**, genus **(B)**, and species levels **(C)**. The amount of microbiota was normalized with that observed before intervention. Statistical difference between the probiotic group and placebo is indicated by a white two-way arrow. Placebo group: T1DM patients receive regular insulin treatment but no additional probiotic supplementation. Probiotic group: T1DM patients receive regular insulin treatment with additional probiotic supplementation.

NGS showed changes in the gut microbiota of the patients before and after intervention; the number of bacteria belonging to genus *Akkermansia*, phylum Verrucomicrobia reduced in both the groups after intervention (**Supplemental Tables S1**, **S2**). Next, we compared and analyzed the difference in the microbiota between the groups. Before the trial, the dominant gut microbiota phyla in both the groups were Actinobacteria, Bacteroidetes, Firmicutes, Proteobacteria, and Verrucomicrobia. Firmicutes accounted for the largest proportion in the probiotic (61.70%) and placebo groups (58.31%), and Actinobacteria had the second largest microbial population in the gut: 24.44% in the probiotic group and 27.05% in the placebo group. Verrucomicrobia accounted for only 4.33% in the probiotic group and 1.78% in the placebo group. However, six months after intervention, the populations of Firmicutes and Actinobacteria did not show significant differences between the groups. Compared with the placebo group, the probiotic group showed a significant difference in the population of Verrucomicrobia (*P* = 0.035) after the intervention (**Figure 1A**).

Changes in the gut microbiota at the genus level were further analyzed after the patients had taken the probiotics for 6 months. *Bifidobacterium* accounted for the major proportion in the probiotic group (24.59%) and in the placebo group (22.52%), *Lactobacillus* accounted for 0.35% in the probiotic group and 0.55% in the placebo group, and *Akkermansia* accounted for 0.28% in the probiotic group and 0.06% in the placebo group. Compared with the placebo group, the probiotic group showed no significant difference in the populations of *Bifidobacterium* and *Lactobacillus* after 6 months of intervention. At the genus level, the population of *Akkermansia* was significantly higher in the probiotic group than in the placebo group after intervention (*P* = 0.035; **Figure 1B**).

NGS analysis of the gut microbiota at the species level revealed that the populations of *B. animalis* (*P* = 0.024), *L. salivarius* (*P* = 0.010), and *Akkermansia* uncultured bacteria (*P* = 0.035) were significantly higher in the probiotic group than in the placebo group after probiotic intervention (**Figure 1C**). Compared with the population before intervention, the population of *L. salivarius* significantly increased after taking probiotics (*P* = 0.001; **Supplemental Table S3**).

### Glycemic Control Declined in 6 Months After Consuming Probiotic Products

The average baseline fasting blood glucose (Glucose AC; mg/dL) before intervention was 185.4 mg/dL in the probiotic group and 172.2 mg/dL in the placebo group, and the average glycated hemoglobin (HbA1c) was 9.3% (78.0 mmol/mol) in the probiotic group and 9.5% (79.9 mmol/mol) in the placebo group **Table 2**). After 6-month probiotic intervention, the average Glucose AC decreased to 161.9 mg/dL (*P* = 0.000) and average glycated hemoglobin decreased to 8.5% (69.7mmol/mol, *P* = 0.000). Six months later, the average glycemic level of the placebo group was similar to the baseline (Glucose AC: 171.5 mg/dL; glycated hemoglobin 9.5% = 80.0 mmol/mol). Three months after stopping the intervention, the glycemic levels of the probiotic group were still lower than the baseline levels (Glucose AC: 163.9 mg/dL, *P* = 0.002; average glycated hemoglobin: 8.9% = 74.1 mmol/mol, *P* = 0.005), whereas the levels of Glucose AC (174.1 mg/dL) and average glycated hemoglobin (9.5% = 80.6 mmol/mol) remained the same as baseline in the placebo group (**Table 2**).

Table 2Glucose AC and HbA1c declined 6 months after consuming probiotic product.

**Table:** 

(a.) Glycemic control.						
	Before-intervention		6-month intervention		3-month after intervention	
	Probiotic group	Placebo group	Probiotic group	Placebo group	Probiotic group	Placebo group
Glucose AC (mg/dl)	185.4 ± 41.5	172.2 ± 62.6	161.9 ± 39.0^###^	171.5 ± 55.5	163.9 ± 39.0^##^	174.1 ± 58.8
HbA1c (%) (mmol/mol)	9.3 ± 0.8 78.0 ± 8.9	9.5 ± 1.9 79.9 ± 21.2	8.5 ± 0.9^###^ 69.7 ± 10.4^###^	9.5 ± 2.1 80.0 ± 22.8	8.9 ± 1.1^##^ 74.1 ± 11.6^##^	9.5 ± 2.2 80.6 ± 24.5

Wilcoxon signed-rank test: when patients in each group were compared with their own before-intervention status, statistical difference was shown as ^##^P < 0.01 and ^###^P < 0.001. Placebo group: T1DM patients receive regular insulin treatment but no additional probiotic supplementation. Probiotic group: T1DM patients receive regular insulin treatment with additional probiotic supplementation.

**Table:** 

(b.) Glycemic control (normalized by individual basal level).				
	6-month intervention/Before-intervention		3-month after intervention/Before-intervention	
	Probiotic group	Placebo group	Probiotic group	Placebo group
Glucose AC (%)	87.5 ± 10.3***	101.5 ± 11.7	89.0 ± 14.4**	103.5 ± 18.3
HbA1c (%)	91.8 ± 5.4***	100.1 ± 7.8	96.1 ± 6.3*	100.7 ± 8.9

Mann–Whitney U test: when patients in the probiotic group were compared with those in the placebo group, statistical difference was shown as *P < 0.05, **P < 0.01 and ***P < 0.001. Placebo group: T1DM patients receive regular insulin treatment but no additional probiotic supplementation. Probiotic group: T1DM patients receive regular insulin treatment with additional probiotic supplementation.

**Figure 2:**
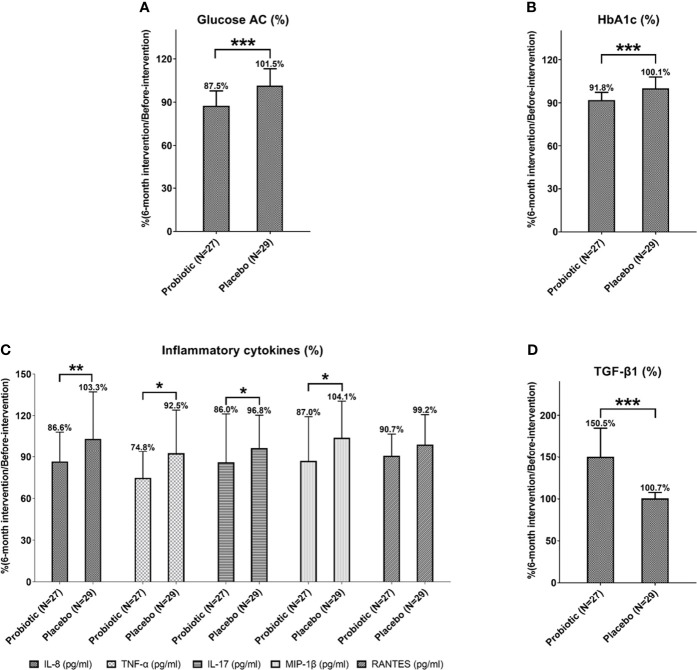
The levels of Glucose AC **(A)**, HbA1c **(B)**, inflammatory cytokines **(C)** and anti-inflammatory cytokine TGF-β1 **(D)** of patients with T1DM after 6-month probiotic intervention were normalized with before-intervention levels and then compared with the placebo group. Statistical differences between probiotic and placebo groups were shown as *P < 0.05, **P < 0.01, and ***P < 0.001. Placebo group: T1DM patients receive regular insulin treatment but no additional probiotic supplementation. Probiotic group: T1DM patients receive regular insulin treatment with additional probiotic supplementation.

The rate of glycemic decline was evaluated by comparing the glycemic control between the probiotic group and placebo group in 6 months after intervention. Data were normalized to the baseline (before-intervention). The average fasting blood glucose rate in the probiotic group significantly dropped to 87.5%, compared with the placebo group, with a level of 101.5% (*P* = 0.000; **Table 2** and **Figure 2A**) and the average glycated hemoglobin (HbA1c) rate decreased to 91.8%, compared with the placebo group, with a rate of 100.1% (*P* = 0.000; **Table 2** and **Figure 2B**). Three months after stopping the intervention, the normalized glycemic levels of the probiotic group were still lower than those of the placebo group (Glucose AC: 89.0%, *P* = 0.001; glycated hemoglobin: 96.1%, *P* = 0.038; **Table 2**). This showed that probiotics can effectively reduce the excessively high blood glucose and glycated hemoglobin in patients with T1DM.

### IL-8, TNF-α, IL-17, MIP-1β, and RANTES Levels Reduced After Taking Probiotics

The serum samples of the patients were collected for further measurements before and after intervention. The results showed that the average levels of the inflammatory cytokines IL-8 (350.4 pg/mL, *P* = 0.000), TNF-α (39.2 pg/mL, *P* = 0.000), IL-17 (19.7 pg/mL, *P* = 0.001), MIP-1β (99.6 pg/mL, *P* = 0.006), and RANTES (401.7 pg/mL, *P* = 0.008) in the probiotic group were significantly decreased after 6 months of probiotic intervention compared with the levels before intervention (**Table 3**). Three months after stopping the intervention, the IL-8 (359.7 pg/mL, *P* = 0.004), TNF-α (45.7 pg/mL, *P* = 0.003), and MIP-1β (98.1 pg/mL, *P* = 0.004) levels were still significantly lower than the before-intervention levels (**Table 3**).

Table 3Analysis of inflammatory and anti-inflammatory cytokines after probiotic intervention.

**Table:** 

(a.) Inflammatory factors						
	Before intervention		6-month intervention		3-month after intervention	
	Probiotic group	Placebo group	Probiotic group	Placebo group	Probiotic group	Placebo group
IL-8 (pg/mL)	398.2 ± 233.4	477.7 ± 222.9	350.4 ± 225.1^###^	467.7 ± 225.3	359.7 ± 233.2^##^	468.6 ± 211.4
TGF-β1 (pg/mL)	5624.2 ± 1984.6	6554.3 ± 1759.3	8034.4 ± 2047.4^###^	6575.2 ± 1739.8	7854.5 ± 2030.7^###^	6482.8 ± 1665.6
TNF-α (pg/mL)	52.5 ± 59.7	54.0 ± 56.8	39.2 ± 47.5^###^	49.3 ± 57.2	45.7 ± 55.7^##^	54.7 ± 59.7
IL-17 (pg/mL)	26.8 ± 34.1	19.5 ± 24.5	19.7 ± 21.3^##^	19.1 ± 24.3	21.6 ± 22.5	18.7 ± 24.5
MIP-1β (pg/mL)	113.9 ± 75.5	130.5 ± 86.9	99.6 ± 76.2^##^	132.3 ± 85.4	98.1 ± 73.7^##^	126.8 ± 78.8
RANTES (pg/mL)	447.9 ± 71.4	411.0 ± 69.0	401.7 ± 75.2^##^	397.2 ± 60.5	428.0 ± 82.0	418.2 ± 54.0

Wilcoxon signed-rank test: when patients in each group were compared with their before-intervention status, statistical difference was shown as ^##^P < 0.01 and ^###^P < 0.001. Placebo group: T1DM patients receive regular insulin treatment but no additional probiotic supplementation. Probiotic group: T1DM patients receive regular insulin treatment with additional probiotic supplementation.

**Table:** 

(b.) Inflammatory factors (normalized to baseline levels).				
	6-month intervention		3-month after intervention	
	Probiotic group	Placebo group	Probiotic group	Placebo group
IL-8 (%)	86.6 ± 21.5**	103.3 ± 33.8	89.6 ± 19.3*	107.0 ± 45.2
TGF-β1 (%)	150.5 ± 34.1***	100.7 ± 7.3	147.0 ± 33.8***	99.4 ± 4.5
TNF-α (%)	74.8 ± 19.1*	92.5 ± 31.6	87.2 ± 21.5	102.4 ± 35.8
IL-17 (%)	86.0 ± 35.3*	96.8 ± 23.6	100.8 ± 49.0	96.4 ± 27.5
MIP-1β (%)	87.0 ± 32.3*	104.1 ± 26.4	85.7 ± 25.4*	101.8 ± 23.2
RANTES (%)	90.7 ± 16.1	99.2 ± 21.5	96.2 ± 15.6	104.9 ± 23.0

Mann–Whitney U test: when patients in the probiotic group were compared with those in the placebo group, statistical difference was shown as *P < 0.05, **P < 0.01, and ***P < 0.001. Placebo group: T1DM patients receive regular insulin treatment but no additional probiotic supplementation. Probiotic group: T1DM patients receive regular insulin treatment with additional probiotic supplementation.

Next, we compared the changes in inflammatory factors between the probiotic group and placebo group. Data were all normalized to the baseline level (before-intervention). The levels of IL-8 (86.6%, *P* = 0.004, TNF-α (74.8%, *P* = 0.018), IL-17 (86.0%, *P* = 0.017), and MIP-1β (87.0%, *P* = 0.029) significantly decreased after 6 months of probiotic intervention (**Figure 2C** and **Table 3**). Three months after stopping intervention, the IL-8 (89.6%, *P* = 0.018) and MIP-1β (85.7%, *P* = 0.016) levels were still significantly lower in the probiotic group than in the placebo group (**Table 3**). Overall, probiotic intervention lowered inflammation levels among patients with T1DM (**Supplemental Figure S2**).

### TGF-β1 Levels Were Elevated After Taking Probiotics

After taking probiotics for 6 months, the level of anti-inflammatory cytokine TGF-β1 significantly increased compared with the before-intervention level (6-month after intervention: 8034.4 pg/mL; before-intervention 5624.2 pg/mL; *P* = 0.000). Three months after stopping the intervention, the TGF-β1 levels (7854.5 pg/mL, *P* = 0.000) were still significantly higher in the probiotic group (**Table 3**). Next, we compared the changes of anti-inflammatory factors between the groups. All data were normalized to the baseline levels (before-intervention). The average normalized TGF-β1 in the probiotic group increased to 150.5%, which was significantly different from the level in the placebo group (*P* = 0.000). The level of TGF-β1 still remained higher (147.0%, *P* = 0.000) than that in the placebo group of 3 months after stopping probiotics (**Figure 2D** and **Table 3**).

### Correlation Between Cytokine Levels and Declined ∆HbA1c

∆HbA1c indicated the HbA1c level after 6-month intervention normalized to the baseline level (before-intervention), ∆HbA1c <1 indicated that the HbA1c level had improved in the intervention period (**Supplemental Figure S3**). In the probiotic group, five inflammatory cytokines (IL-8, TNF-α, IL-17, MIP-1β and RANTES) were down-regulated together with declined ∆HbA1c level after consuming probiotic strains for 6 months. The anti-inflammatory cytokines (TGF-β1) was up-regulated together with declined ∆HbA1c level after consuming probiotic strains for 6 months.

In the placebo group, 47.4% (N = 9) of the patients showed improved IL-8 levels along with improved ∆HbA1c levels, 71.4% (N = 10) of the patients showed improved TGF-β1 levels together with improved ∆HbA1c levels, 38.9% (N = 7) of the patients showed improved TNF-α levels together with improved ∆HbA1c levels, 47.1% (N = 8) of the patients showed improved IL-17 levels together with improved ∆HbA1c levels, 50.0% (N = 7) of the patients showed improved MIP-1β levels, and 56.3% (N = 9) of the patients showed improved RANTES levels. This result indicated that consumption of probiotics could help improve the inflammatory response and reduce excessive HbA1c in patients with T1DM (**Supplemental Figure S3**). In addition, the majority of patients with T1DM showed improved ∆Glucose AC along with reduced inflammatory cytokine levels after probiotic intervention (**Supplemental Figure S4**).

## Discussion

Glycemic management is a critical issue in diabetes (30). In addition to medical interventions (31), studies have investigated the role of probiotics in regulating hyperglycemia in diabetes mellitus (32). Several studies have reported about the effects of probiotics on downregulation of glycemia in patients with T2DM; Asemi et al. discovered that multispecies probiotics, namely *L. acidophilus, L. rhamnosus*, *L. casei*, *L. bulgaricus*, *B. longum*, and *Streptococcus thermophilus* could prevent the rise of fasting plasma glucose (FPG), reduce serum hs-C-reactive protein, and elevate plasma total glutathione in T2DM (33). Ejtahed et al. revealed that probiotic yogurt containing *L. acidophilus* and *B. lactis* could regulate FPG, hemoglobin A1c (HbA1c), and antioxidant levels in patients with T2DM (34). In addition, symbiotic bread containing viable and heat-treated *L. sporogenes* helped reduce serum triacylglycerol and very-low-density lipoprotein levels, while improving high-density lipoprotein levels in patients with T2DM (35). However, few studies have reported on their effects on T1DM. Whether probiotic supplementation altered gut microbiota profile in T1DM is still unclear. At present study, it is suggested that probiotics plus regular insulin treatment would present steadier control of Glucose AC level and HbA1c than insulin treatment only (placebo group) (**Table 2**).

In this study, NGS demonstrated that consumption of probiotic *L. salivarius* subsp. *salicinius* AP-32, *L. johnsonii* MH-68, and *B. animalis* subsp. *lactis* CP-9 could successfully enrich the population of these gut microbiota, especially in the species of *Bifidobacterium animalis, Lactobacillus salivarius* and *Akkermansia muciniphila* (**Figure 1**). The colonized beneficial microbiota may play an important role in slowing down the levels of Glucose AC and HbA1c in patients with T1DM (**Table 2**). The Oral glucose tolerance test (OGTT) is a reliable method for measuring glycemic levels (36). However, the WHO mentioned several shortcomings of OGTT test in clinical practice including inconvenience, greater cost and less reproducibility (37). Thus, the HbA1c and Glucose AC levels were selected as glycemic biomarkers of practical clinical practice at this study.

Furthermore, abundance of *Akkermansia muciniphila* has been reported to negatively correlate with HbA1c levels (38). Studies have revealed that obese children (above 3 years of age) had lower levels of *A. muciniphila* in their gut microbiota (39). Moreover, *A. muciniphila* can restore the gut barrier at the mucous layer, facilitate lipid metabolism, reduce fat mass accumulation, and reduce the incidence of fatty liver and hypercholesterol (40). This may account for the higher distribution of *A. muciniphila* among semi-supercentenarians (41). In our study, patients with T1DM who received the novel probiotic product slowed down the declined rate of *Akkermansia* uncultured bacterium in their gut, compared with patients who received the placebo. The molecular mechanism underlying the modulation of glycemic levels by the colonized microbiota warrants further investigation.

Previous clinical studies have discovered the relationship between human immunity and probiotic consumption. Groele et al. found that *L. rhamnosus* GG and *B. lactis* Bb12 had several advantages in T1DM, including improvement of the gut mucosal barrier, regulation of local and systemic immune responses, reduction of the risk of autoimmunity, restriction of the growth of pathogens, and preservation of β-cell function (42). Another clinical study involving 42 healthy individuals reported that *L. johnsonii* N6.2 could have beneficial effects on T1DM by regulating the kynurenine:tryptophan ratio, indoleamine 2,3-dioxygenase pathway, circulatory effector Th1 cells, and cytotoxic CD8^+^ T cells (43). While previous researches might have explored the benefits of probiotics to T1DM, this is a pioneer study to include post-experimental follow-ups, where the persistence effect of immunomodulation by proinflammatory cytokine diminution and anti-inflammatory cytokine accretion was observed even 3 months after discontinuation of the probiotics.

Gut microbiome and their metabolites may exert function by affecting intestinal permeability, molecular mimicry, and modulating immune system (44). In this study, we revealed the ability of *L. salivarius* subsp. *salicinius* AP-32, *L. johnsonii* MH-68, and *B. animalis* subsp. *lactis* CP-9 to downregulate the immune-related inflammatory cytokines IL-8, TNF-α, IL-17, MIP-1β, and RANTES and upregulate the anti-inflammatory cytokine TGF-β1 (**Table 3** and **Supplemental Figure S2**). Cytokines and chemokines have previously been implicated in the pathogenesis of T1DM (45), including IL-8 (46), RANTES (47), MIP-1β (48), TNF-α (49), and IL-17 (50). On the other hand, TGF-β secreted by regulatory T cells (Tregs) can suppress inflammation (51). Tregs help to prevent the development of T1DM; they delay the onset of T1DM by secretion of TGF-β (52). Next, the correlation between blood sugar levels, cytokine levels and the change of intestinal flora after treating probiotics were analyzed by Spearman rank-order correlation test. The results suggested that *Actinobacteria, Firmicutes, Bifidobacterium bifidum, Bacteoid fragilis*, and *Lactobacillus salivarius* showed the negative correlation with inflammatory cytokine TNF-α, IL-8, RANTES, MIP-1β, and Glucose AC, respectively. A negative correlation means that as one variable increases, the other tends to decline. Besides, *Lactobacillus salivarius* presented positive correlation with the anti-inflammatory cytokine TGF-β1 (**Supplemental Figure S5**).

Our results also demonstrated the correlation between improved cytokine levels and declined glycemic rate among patients with T1DM after probiotic intervention (**Supplemental Figures S3**, **S4**). Previous studies discovered that elevation in the concentrations of inflammatory cytokines such as TNF-α associated with glycemic control and cardiovascular risk factors among patients with T1DM (53). The results of this study suggested that probiotics may play an important role in reducing immune inflammatory cytokines and glycemic control in patients with T1DM. However, the mechanism by which the elevated inflammatory cytokines stimulate blood glucose (through which glucose transporters or insulin receptors) among patients with T1DM warrants further investigation.

The limitation of our study lies in the lack of knowledge to how the probiotics modulate the immunity to improve blood sugar. Firstly, while a correlation between our probiotics and improved blood sugar was observed, more work might be required to elucidate the underlying mechanism in order to explain for the causality. Secondly, as the current study utilizes a multi-strain supplement to make use of the synergic effect, more studies might be needed to clarify the individual effects of the strains contained in our probiotics. Thirdly, changes of c-peptide (54) and beta-cells (55) levels by probiotic intervention should be detected. Finally, the NGS sequencing method used in the article was to identify gut microbial bacteria by sequencing the V3-V4 region of 16S rRNA (56) with the annotations of SILVA database. The sequencing method still has some limitations in microbial classification. Therefore, the qPCR method with specific primers should be performed for further validating microbial taxonomy in the future.

## Conclusion

The results of this study showed that conventional insulin treatment plus mixed probiotics strains of *L. salivarius* subsp. *salicinius* AP-32, *L. johnsonii* MH-68, and *B. animalis* subsp. *lactis* CP-9 showed better treatment outcome than insulin injection only (the placebo group). It’s suggested that insulin treatment plus probiotic supplementation could enrich the population of beneficial gut microbiota in the gut (*Bifidobacterium animalis, Lactobacillus salivarius* and *Akkermansia muciniphila*), improve the glycemic control (Glucose AC and HbA1c), reduce the levels of inflammation-related cytokines (IL-8, TNF-α, IL-17, MIP-1β, and RANTES) and increase the levels of the anti-inflammatory cytokine TGF-β1 in patients with T1DM. The results of this study render a prospective therapeutic option for clinical T1DM treatment.

## Data Availability Statement

The datasets presented in this study can be found in online repositories. The names of the repository/repositories and accession number(s) can be found below: “NCBI with Bioproject ID PRJNA798680 (https://www.ncbi.nlm.nih.gov/bioproject/PRJNA798680/).

## Ethics Statement

This clinical trial complied with the Declaration of Helsinki, and it was reviewed and approved by China Medical University & Hospital Research Ethics Committee (CMUH107-REC2-036).

## Author Contributions

C-HW: conceptualization, reviewing, and editing. H-RY: reviewing and editing. W-LL: reviewing and editing. H-HH: supervision and project design. W-YL: original drafting. Y-WK: methodology and data analysis. Y-YH: methodology, data visualization, and project administration. S-YT: reviewing and editing. H-CL: supervision, reviewing, and editing. All authors contributed to the article and approved the submitted version.

## Funding

This study was supported by grants from China Medical University Hospital (DMR-108-048, DMR-109-052 and DMR-110-209) and Asia University Hospital (ASIA-108-51010).

## Conflict of Interest

Author H-HH, W-YL, Y-WK, Y-YH, and S-YT were employed by Bioflag Biotech Co., Ltd.

The remaining authors declare that the research was conducted in the absence of any commercial or financial relationships that could be construed as a potential conflict of interest.

## Publisher’s Note

All claims expressed in this article are solely those of the authors and do not necessarily represent those of their affiliated organizations, or those of the publisher, the editors and the reviewers. Any product that may be evaluated in this article, or claim that may be made by its manufacturer, is not guaranteed or endorsed by the publisher.
