# Supplementary material for: Adjuvant Probiotics of Lactobacillus salivarius subsp. salicinius AP-32, L. johnsonii MH-68, and Bifidobacterium animalis subsp. lactis CP-9 Attenuate Glycemic Levels and Inflammatory Cytokines in Patients With Type 1 Diabetes Mellitus
Source: Front Endocrinol (Lausanne). 2022 Mar 1;13:754401. doi: 10.3389/fendo.2022.754401 (PMC8921459; doi:10.3389/fendo.2022.754401)
Supplement: Supplementary file 1 [file DataSheet_1.zip › Supplemental Figure captions.docx]

**Supplemental figure captions**

**Supplemental Figure S1** Flow diagram of recruiting experimental participants.

**Supplemental Figure S2** Changes in inflammatory and anti-inflammatory cytokines. After 6 months of taking probiotics or placebo, 81.5% (N=22) of the patients showed decreased serum IL-8 concentration in the probiotic group, whereas 65.5% (N=19) decreased IL-8 in the placebo group. 96.3% (N=26) of the patients showed increased TGF-β1in probiotic group, whereas placebo group accounted for 48.3% (N=14). 96.3% (N=26) of the patients showed down-regulated TNF-α concentration in probiotic group, whereas placebo group accounted for 62.1% (N=18). 77.8% (N=21) of the patients showed decreased IL-17 levels in probiotic group, whereas 58.6% (N=17) in placebo group. 74.1% (N=20) of the patients showed decreased MIP-1β in the probiotic group, whereas 48.3% (N=14) in placebo. 70.4% (N=19) of the patients showed decreased RANTES in the probiotic group, whereas the placebo group accounted for 55.2% (N=16). This result indicates that patients with type 1 diabetes who take probiotics can improve the inflammatory response in the body and increase the anti-inflammatory effect. All data were normalized to before-intervention levels (%).

**Supplemental Figure S3** Correlation between inflammatory and anti-inflammatory cytokines and ∆HbA1c. After 6 months of taking probiotics or placebo, the glycosylated hemoglobin (∆HbA1c) of patients with decreased inflammatory cytokines and increased anti-inflammatory (TGF-β1) cytokine levels was analyzed. All data were normalized to before-intervention levels (%).

**Supplemental Figure S4** Correlation between inflammatory and anti-inflammatory cytokines and ∆Glucose AC. After 6 months of taking probiotics or placebo, 86.4% (N=19) of patients showed improved ∆Glucose AC with decreased IL-8 level in probiotic group, whereas the placebo group accounted for 42.1% (N=8). 88.5% (N=23) of patients showed improved ∆Glucose AC with increased TGF-β1 in probiotic group, whereas the placebo group accounted for 50.0% (N=7). 88.5% (N=23) of patients showed improved ∆Glucose AC with decreased TNF-α in probiotic group, whereas placebo group accounted for 50.0% (N=9). 90.5% (N=19) of patients showed improved ∆Glucose AC with decreased IL-17 in probiotic group, whereas placebo group accounted for 41.2% (N=7). 90.0% (N=18) of patients showed improved ∆Glucose AC with decreased MIP-1β in probiotic group, whereas placebo group accounted for 57.1% (N=8). 89.5% (N= 17) of patients showed improved ∆Glucose AC with decreased RANTES in probiotic group, whereas the placebo group accounted for 50.0% (N=8). This result suggested that patients with T1DM improved the inflammatory levels and reduced the excessive fasting blood sugar after taking probiotic product. All data were normalized to before-intervention levels (%).

**Supplemental Figure S5** The correlation between blood sugar levels, cytokine levels and the change of intestinal flora after treating probiotics were analyzed by Spearman rank-order correlation test. Spearman rank-order correlation test was performed by using SPSS 12 (IBM, USA), where the value R = 1 means a perfect positive linear correlation. P < 0.05 was considered statistically significant. A negative correlation means that as one variable increases, the other tends to decline. A positive correlation indicates that as one variable increases, the other variable also tends to increase.
