# Supplementary material for: Adjuvant Probiotics of Lactobacillus salivarius subsp. salicinius AP-32, L. johnsonii MH-68, and Bifidobacterium animalis subsp. lactis CP-9 Attenuate Glycemic Levels and Inflammatory Cytokines in Patients With Type 1 Diabetes Mellitus
Source: Front Endocrinol (Lausanne). 2022 Mar 1;13:754401. doi: 10.3389/fendo.2022.754401 (PMC8921459; doi:10.3389/fendo.2022.754401)
Supplement: Supplementary file 1 [file DataSheet_1.zip › Supplemental Tables S1-S3.docx]

**Supplemental Table S1** NGS analysis of gut microbiota change in phylum level.

| **Within-group** | **Probiotics** | | | **Placebo** | | |
| --- | --- | --- | --- | --- | --- | --- |
| **Group** | **Before-intervention** | **Intervention** | **p value** | **Before-intervention** | **Intervention** | **p value** |
| *Acidobacteria* | 0.00013% | 0.00000% | 0.1797 | 0.00004% | 0.00000% | 0.3173 |
| *Actinobacteria* | 24.44074% | 32.27232% | 0.0615 | 27.05445% | 30.86657% | 0.1909 |
| *Bacteroidetes* | 6.63239% | 6.24061% | 0.7982 | 10.10704% | 6.93985% | 0.3949 |
| *Chlamydiae* | 0.00117% | 0.00000% | 0.3173 | 0.00000% | 0.00000% | 1.0000 |
| *Chloroflexi* | 0.00018% | 0.00015% | 0.4990 | 0.00009% | 0.00000% | 0.3173 |
| *Cyanobacteria* | 0.00276% | 0.00097% | 0.2094 | 0.02723% | 0.00221% | 0.6909 |
| *DeinococcusThermus* | 0.00126% | 0.00000% | 0.3173 | 0.00009% | 0.00000% | 0.3173 |
| *Epsilonbacteraeota* | 0.00005% | 0.00027% | 0.2733 | 0.00074% | 0.00178% | 0.6121 |
| *Firmicutes* | 61.70741% | 57.34108% | 0.1919 | 58.31825% | 59.34926% | 0.6204 |
| *Fusobacteria* | 0.42057% | 0.05012% | 0.1842 | 0.11301% | 0.04415% | 0.5862 |
| *Gemmatimonadetes* | 0.00009% | 0.00008% | 0.6547 | 0.00000% | 0.00000% | 1.0000 |
| *Patescibacteria* | 0.01213% | 0.01569% | 0.9250 | 0.01002% | 0.01193% | 0.3130 |
| *Planctomycetes* | 0.00012% | 0.00000% | 0.3173 | 0.00000% | 0.00007% | 0.3173 |
| *Proteobacteria* | 2.20186% | 3.54615% | 0.1658 | 2.35720% | 2.49555% | 0.8093 |
| *Synergistetes* | 0.00552% | 0.00612% | 0.4990 | 0.00732% | 0.00296% | 0.2604 |
| *Tenericutes* | 0.00085% | 0.00062% | 0.4652 | 0.00005% | 0.00009% | 0.3173 |
| *Verrucomicrobia* | 4.33134% | 0.28165% | 0.0037** | 1.78856% | 0.06118% | 0.0186* |

Wilcoxon signed-rank test: *P < 0.05, **P < 0.01.

**Supplemental Table S2** NGS analysis of gut microbiota change in genus level.

| **Within-group** | **Probiotics** | | | **Placebo** | | |
| --- | --- | --- | --- | --- | --- | --- |
| **Group** | **Before-intervention** | **Intervention** | **p value** | **Before-intervention** | **Intervention** | **p value** |
| *Bifidobacterium* | 17.25890% | 24.59772% | 0.0875 | 19.47345% | 22.52064% | 0.1996 |
| *Eggerthella* | 0.77857% | 0.81041% | 0.9893 | 0.45483% | 0.48624% | 0.8489 |
| *Bacteroides* | 5.68080% | 5.39824% | 0.6571 | 8.97449% | 5.83004% | 0.3158 |
| *Prevotella* | 0.00437% | 0.00943% | 0.6529 | 0.01488% | 0.00605% | 0.5901 |
| *Prevotella 2* | 0.00119% | 0.00000% | 0.3173 | 0.00563% | 0.00164% | 0.3173 |
| *Prevotella 6* | 0.00165% | 0.00051% | 0.7150 | 0.00364% | 0.00037% | 0.1731 |
| *Prevotella 7* | 0.00013% | 0.00024% | 0.6547 | 0.00650% | 0.00000% | 0.1797 |
| *Prevotella 9* | 0.03259% | 0.00073% | 0.7150 | 0.07647% | 0.21061% | 0.017* |
| *Alistipes* | 0.24591% | 0.30143% | 0.7089 | 0.24217% | 0.24751% | 0.8484 |
| *Bacillus* | 0.00009% | 0.00022% | 0.2733 | 0.00004% | 0.00004% | 0.6547 |
| *Staphylococcus* | 0.00333% | 0.00242% | 0.5540 | 0.00256% | 0.00193% | 0.4703 |
| *Enterococcus* | 0.67620% | 0.55509% | 0.4073 | 0.20245% | 0.67757% | 0.9090 |
| *Lactobacillus* | 0.32421% | 0.35133% | 0.7103 | 1.01629% | 0.55794% | 0.1005 |
| *Lactococcus* | 0.03262% | 0.07360% | 0.7317 | 0.04517% | 0.05716% | 0.4386 |
| *Streptococcus* | 6.64563% | 3.73171% | 0.1500 | 3.76200% | 4.55701% | 0.2376 |
| *Clostridium sensu stricto 1* | 0.71799% | 0.65164% | 0.9036 | 0.45845% | 0.30531% | 0.8291 |
| *Clostridium sensu stricto 2* | 0.00015% | 0.00005% | 0.6547 | 0.00000% | 0.00000% | 1.0000 |
| *Clostridium sensu stricto 6* | 0.00000% | 0.00019% | 0.3173 | 0.00000% | 0.00000% | 1.0000 |
| *Eubacterium* | 0.04989% | 0.07677% | 0.7299 | 0.08409% | 0.05755% | 0.4460 |
| *Clostridioides* | 0.00278% | 0.01066% | 0.4631 | 0.01595% | 0.01263% | 0.4990 |
| *Ruminococcus 1* | 0.36442% | 0.31659% | 0.3011 | 0.67338% | 0.60076% | 0.4237 |
| *Ruminococcus 2* | 3.69520% | 3.71299% | 0.2758 | 4.41697% | 3.95206% | 0.3158 |
| *Veillonella* | 0.11797% | 0.11669% | 0.7775 | 0.63927% | 0.27910% | 0.8689 |
| *Escherichia-Shigella* | 1.74201% | 2.62619% | 0.3674 | 1.40771% | 1.69853% | 0.8291 |
| *Klebsiella* | 0.18690% | 0.18742% | 0.5633 | 0.23599% | 0.37075% | 0.4386 |
| *Proteus* | 0.00005% | 0.00000% | 0.3173 | 0.00000% | 0.00012% | 0.3173 |
| *Akkermansia* | 4.33134% | 0.28165% | 0.0037** | 1.78856% | 0.06118% | 0.0186* |

Wilcoxon signed-rank test: *P < 0.05, **P < 0.01.

**Supplemental Table S3** NGS analysis of gut microbiota change in species level.

| **Within-group (reads %)** | **Probiotics** | | | **Placebo** | | |
| --- | --- | --- | --- | --- | --- | --- |
| **Group** | **Before-intervention** | **Intervention** | **p value** | **Before-intervention** | **Intervention** | **p value** |
| *Bifidobacterium adolescentis* | 0.00455% | 0.00867% | 0.2959 | 0.00745% | 0.00621% | 0.2641 |
| *Bifidobacterium animalis* | 0.00659% | 0.01662% | 0.3139 | 0.00877% | 0.00022% | 0.4652 |
| *Bifidobacterium bifidum* | 0.44139% | 1.28628% | 0.1997 | 1.04834% | 0.94662% | 0.8583 |
| *Bifidobacterium breve* | 0.01504% | 0.06165% | 0.2414 | 0.02534% | 0.05250% | 0.0318* |
| *Bifidobacterium kashiwanohense* | 0.00738% | 0.00979% | 0.4997 | 0.00625% | 0.00924% | 0.0702 |
| *Bifidobacterium longum* subsp. *longum* | 1.39367% | 1.61240% | 0.9464 | 1.43588% | 1.68017% | 0.1742 |
| *Bifidobacterium pseudocatenulatum* | 0.04312% | 0.06773% | 0.0814 | 0.04225% | 0.05352% | 0.3382 |
| *Bacteroides fragilis* | 0.00028% | 0.00053% | 0.6547 | 0.00000% | 0.00000% | 1.0000 |
| *Bacillus amyloliquefaciens* | 0.00005% | 0.00012% | 0.6547 | 0.00000% | 0.00004% | 0.3173 |
| *Staphylococcus sciuri* | 0.00004% | 0.00012% | 0.2850 | 0.00015% | 0.00000% | 0.3173 |
| *Enterococcus durans* | 0.00362% | 0.00262% | 0.6121 | 0.00084% | 0.00560% | 0.3454 |
| *Enterococcus faecalis* | 0.08769% | 0.00586% | 0.2209 | 0.02112% | 0.01017% | 0.5732 |
| *Enterococcus faecium* | 0.01230% | 0.00967% | 0.9528 | 0.00309% | 0.01585% | 0.4603 |
| *Enterococcus gallinarum* | 0.00743% | 0.00813% | 0.8767 | 0.00760% | 0.00645% | 0.4204 |
| *Lactobacillus casei* | 0.00743% | 0.00179% | 0.3454 | 0.01118% | 0.00018% | 0.3270 |
| *Lactobacillus fermentum* | 0.00115% | 0.00030% | 0.5930 | 0.00264% | 0.02078% | 0.4008 |
| *Lactobacillus rhamnosus* | 0.07733% | 0.02661% | 0.7299 | 0.17186% | 0.00359% | 0.4703 |
| *Lactobacillus salivarius* | 0.01578% | 0.05450% | 0.0014** | 0.03977% | 0.03870% | 0.6858 |
| *Lactococcus garvieae* subsp. *garvieae* | 0.01799% | 0.05187% | 0.7439 | 0.03507% | 0.02118% | 0.9032 |
| *Lactococcus lactis* | 0.00004% | 0.00022% | 0.2850 | 0.00000% | 0.00034% | 0.1797 |
| *Streptococcus agalactiae* | 0.01058% | 0.00220% | 0.2411 | 0.01083% | 0.00270% | 0.5147 |
| *Streptococcus gallolyticus* subsp. *macedonicus* | 0.01808% | 0.00374% | 0.0712 | 0.00735% | 0.00816% | 0.9594 |
| *Streptococcus mutans* | 0.00112% | 0.00160% | 0.8313 | 0.00127% | 0.00107% | 0.4380 |
| *Streptococcus parauberis* | 0.01972% | 0.00019% | 0.2367 | 0.00032% | 0.00088% | 0.4446 |
| *Streptococcus salivarius* subsp. *thermophilus* | 0.00230% | 0.00696% | 0.6813 | 0.00193% | 0.00459% | 0.7943 |
| *Streptococcus sobrinus* | 0.02660% | 0.01225% | 0.1159 | 0.00381% | 0.00097% | 0.1441 |
| *Clostridium* subsp. *CL-2* | 0.00151% | 0.00005% | 0.2850 | 0.00262% | 0.00013% | 0.6547 |
| *Eubacterium hallii* | 2.13815% | 2.44122% | 0.4750 | 2.21958% | 2.55154% | 0.4385 |
| *Clostridioides difficile* | 0.00278% | 0.01045% | 0.4631 | 0.01573% | 0.01257% | 0.4990 |
| *Escherichia coli* | 0.00761% | 0.01578% | 0.2627 | 0.01300% | 0.00756% | 0.3458 |
| *Klebsiella pneumoniae* | 0.01665% | 0.02056% | 0.5901 | 0.01325% | 0.05320% | 0.1443 |
| *Akkermansia* uncultured bacterium | 4.33134% | 0.28165% | 0.0037** | 1.78856% | 0.06118% | 0.0186* |

Wilcoxon signed-rank test: *P < 0.05, **P < 0.01.
