# Supplementary figures and images for: Adjuvant Probiotics of Lactobacillus salivarius subsp. salicinius AP-32, L. johnsonii MH-68, and Bifidobacterium animalis subsp. lactis CP-9 Attenuate Glycemic Levels and Inflammatory Cytokines in Patients With Type 1 Diabetes Mellitus
Source: Front Endocrinol (Lausanne). 2022 Mar 1;13:754401. doi: 10.3389/fendo.2022.754401 (PMC8921459; doi:10.3389/fendo.2022.754401)

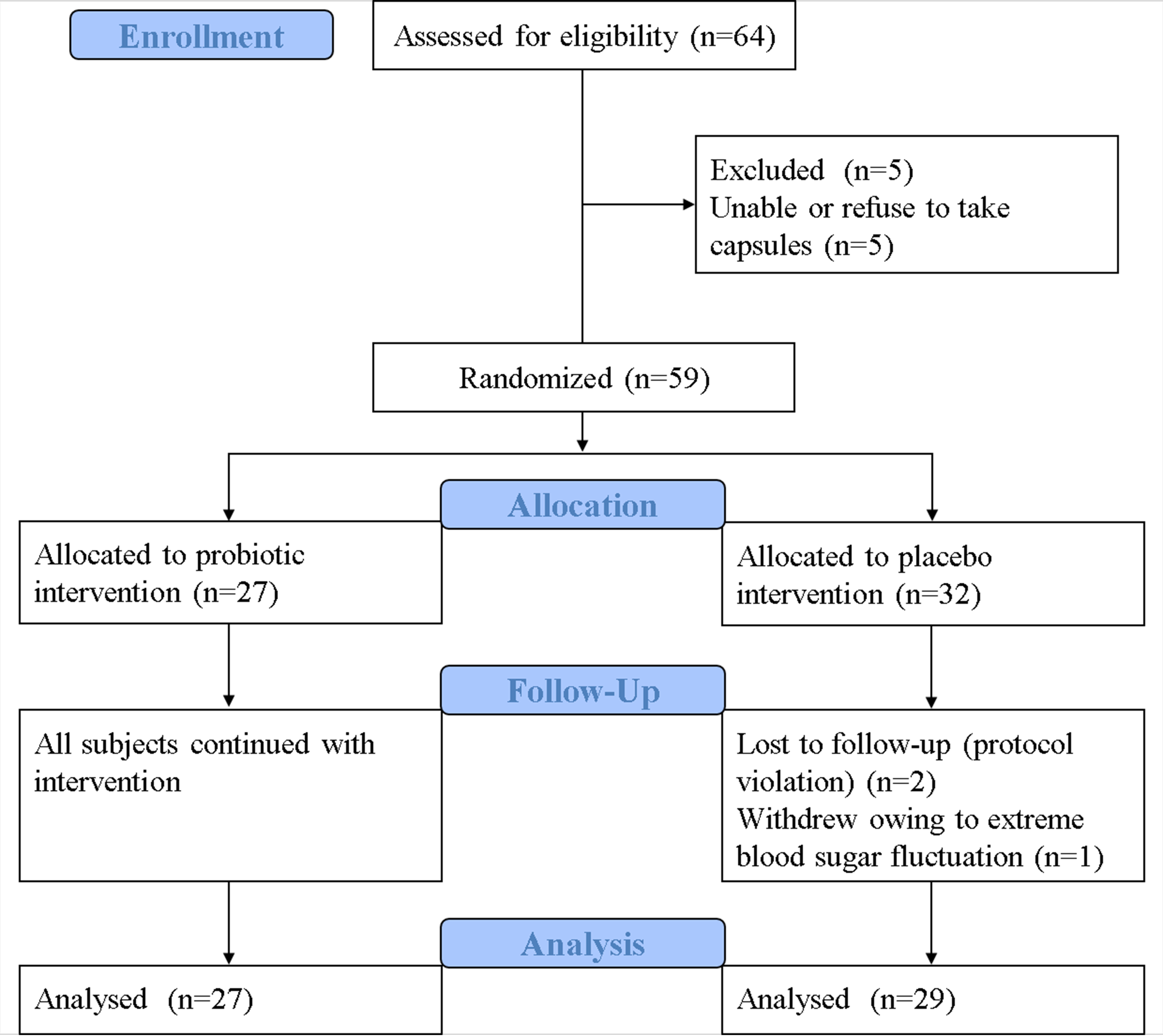

Supplement: Supplementary file 1 [file DataSheet_1.zip › Supplemental Figure S1.TIF]

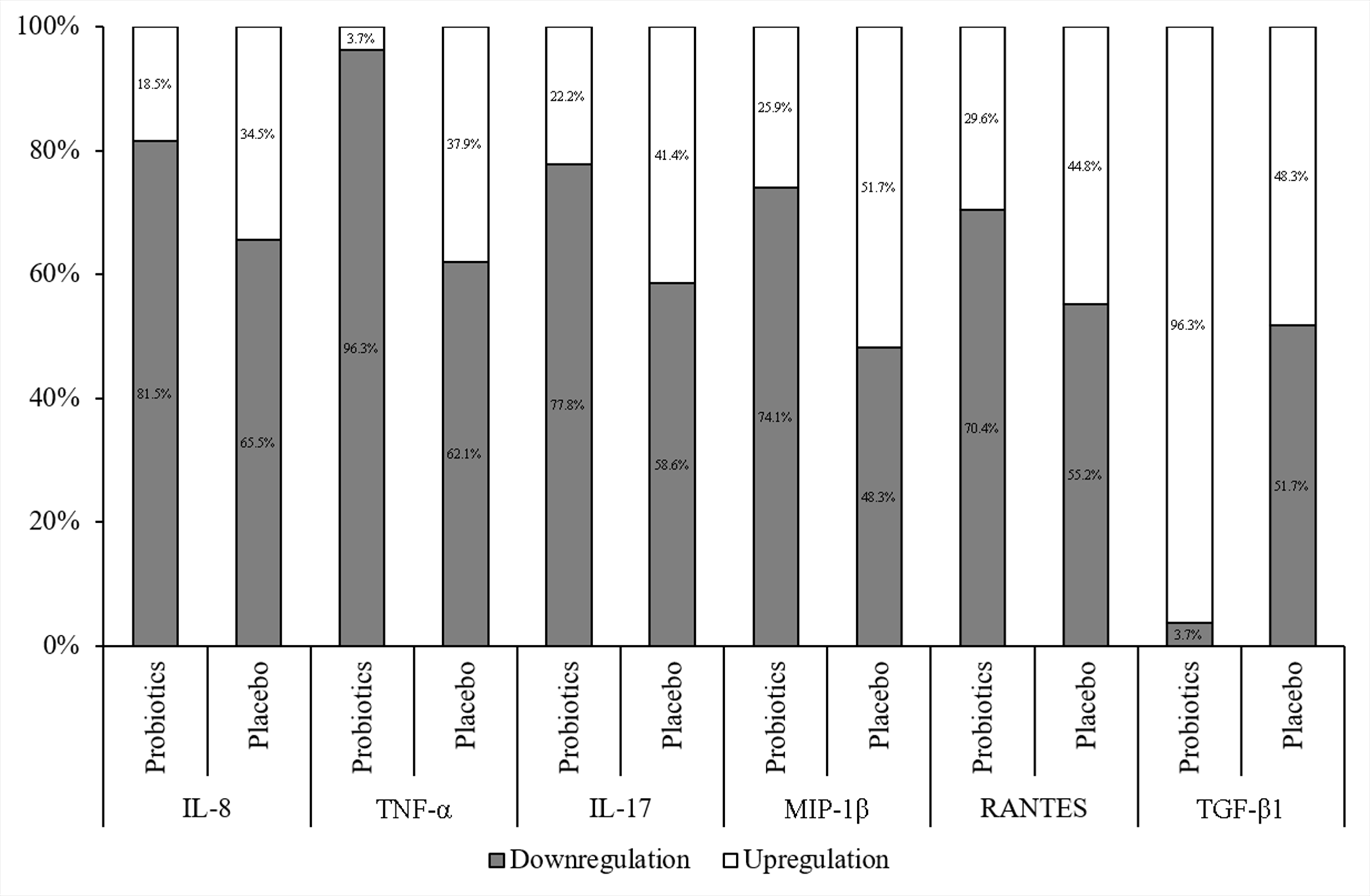

Supplement: Supplementary file 1 [file DataSheet_1.zip › Supplemental Figure S2.TIF]

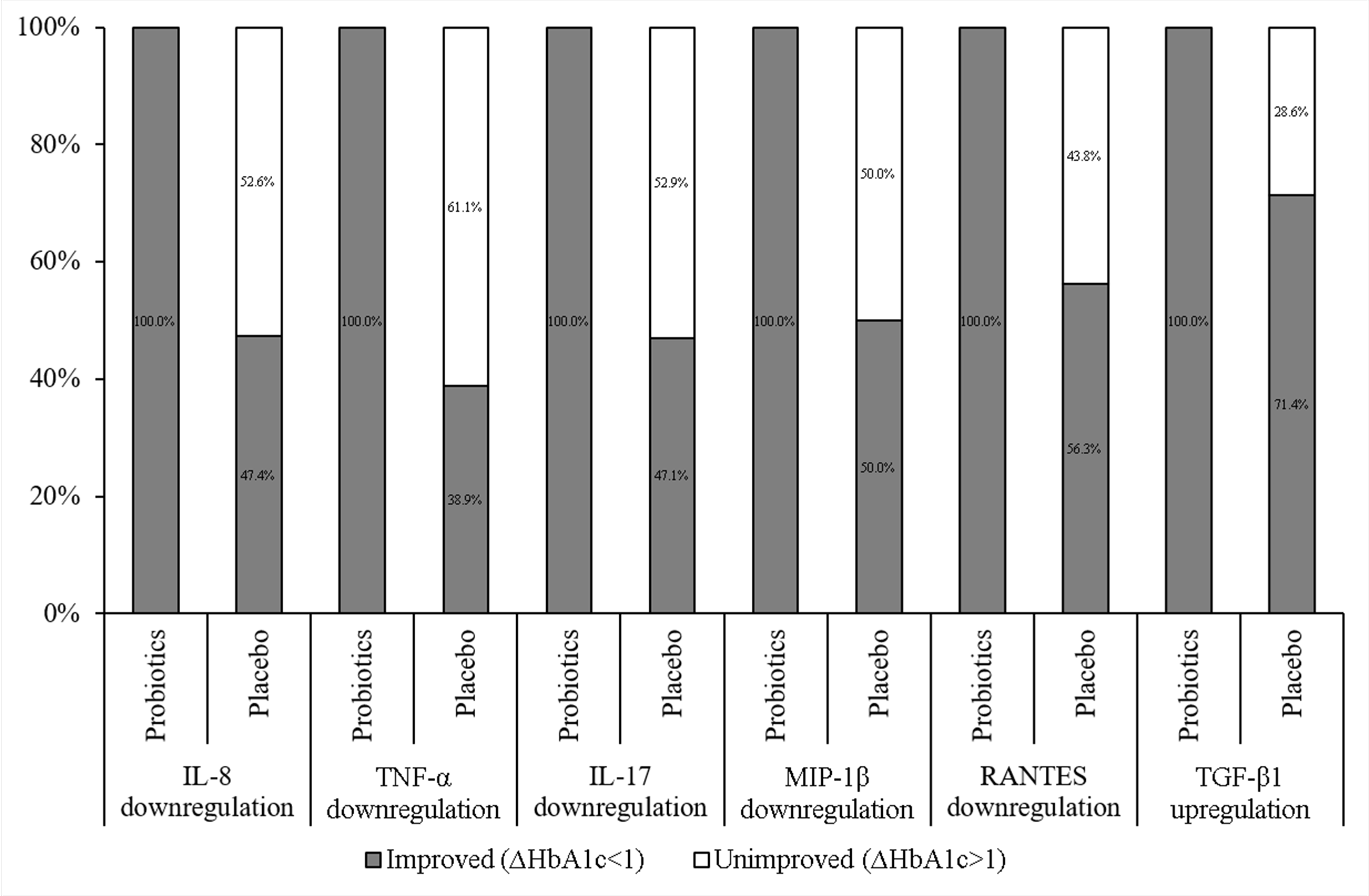

Supplement: Supplementary file 1 [file DataSheet_1.zip › Supplemental Figure S3.TIF]

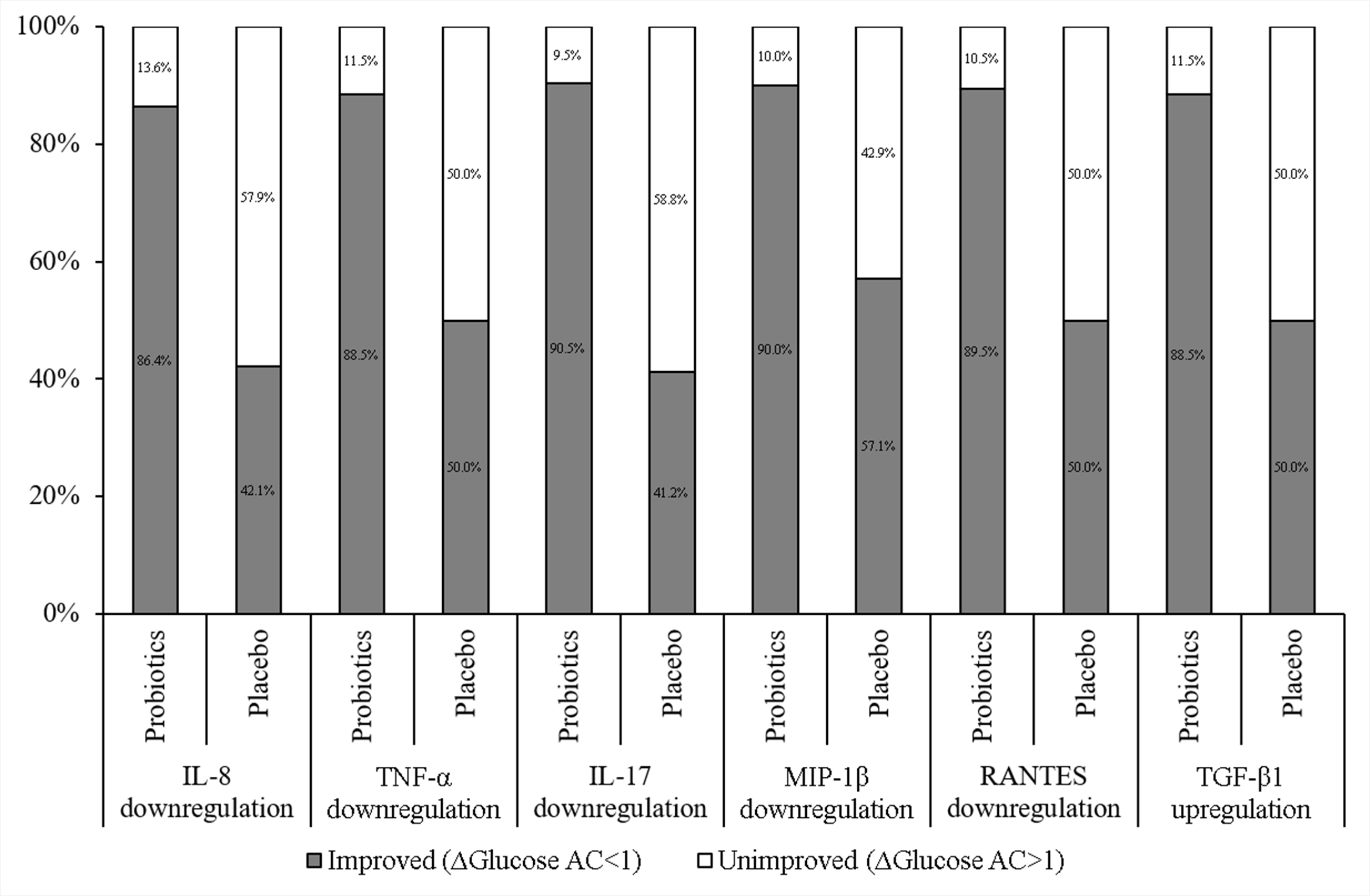

Supplement: Supplementary file 1 [file DataSheet_1.zip › Supplemental Figure S4.tif]

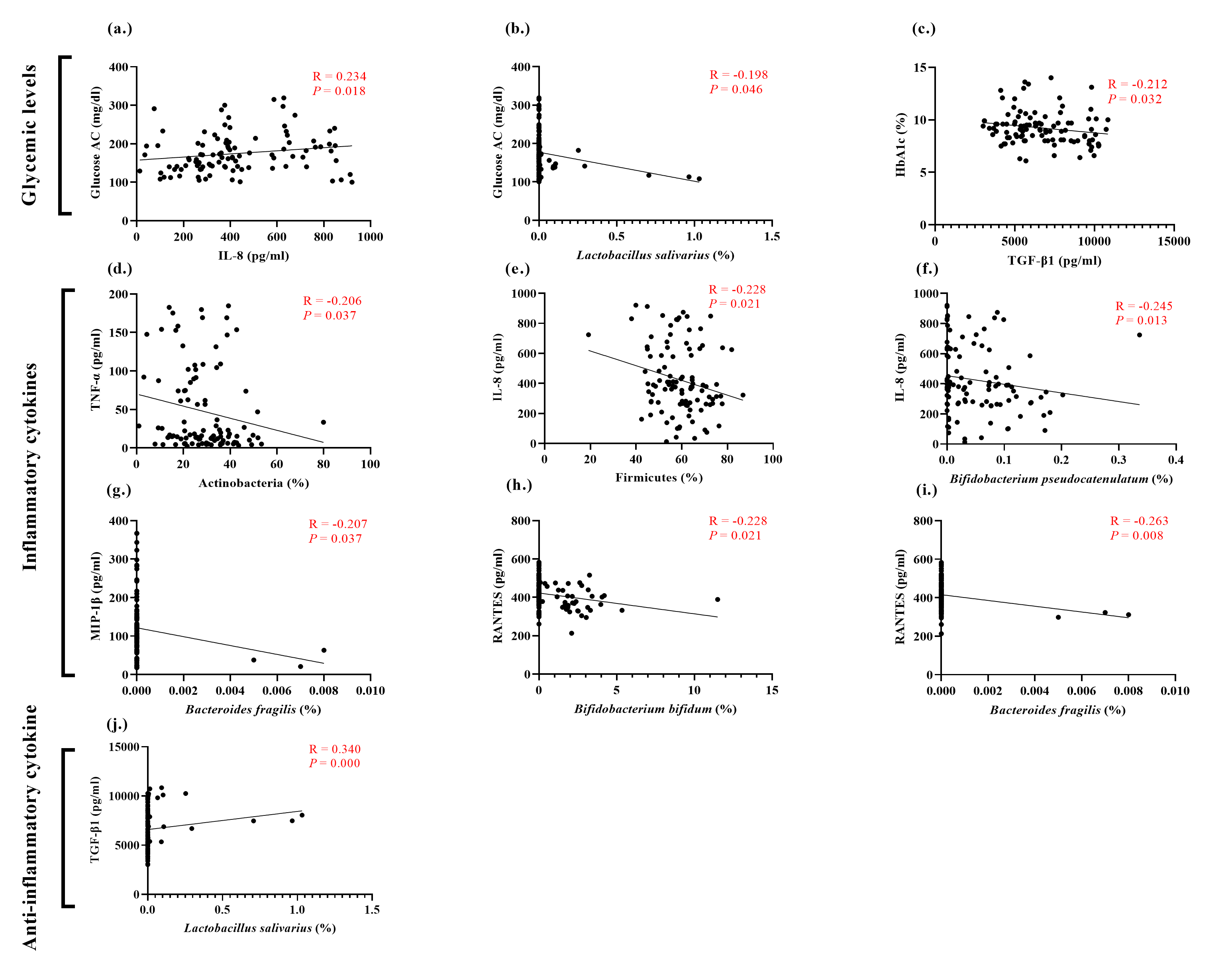

Supplement: Supplementary file 1 [file DataSheet_1.zip › Supplemental Figure S5.TIF]
